# Expedient assessment of post-infarct remodeling by native cardiac magnetic resonance imaging in mice

**DOI:** 10.1038/s41598-021-91096-4

**Published:** 2021-06-02

**Authors:** Cajetan Immanuel Lang, Praveen Vasudevan, Piet Döring, Ralf Gäbel, Heiko Lemcke, Tobias Lindner, Gustav Steinhoff, Bernd Joachim Krause, Brigitte Vollmar, Felix G. Meinel, Seyrani Yücel, Alper Öner, Hüseyin Ince, Robert David

**Affiliations:** 1 Department of Cardiology, Rostock University Medical Center, Rostock, Germany; 2 Department of Cardiac Surgery, Rostock University Medical Center, Rostock, Germany; 3 Department of Life, Light and Matter, University of Rostock, Rostock, Germany; 4 Core Facility Multimodal Small Animal Imaging, Rostock University Medical Center, Rostock, Germany; 5 Department of Nuclear Medicine, Rostock University Medical Center, Rostock, Germany; 6 Rudolf-Zenker-Institute for Experimental Surgery, Rostock University Medical Center, Rostock, Germany; 7 Institute of Diagnostic and Interventional Radiology, Rostock University Medical Center, Rostock, Germany

**Keywords:** Preclinical research, Experimental models of disease, Cardiovascular biology, Heart failure

## Abstract

Novel therapeutic strategies aiming at improving the healing process after an acute myocardial infarction are currently under intense investigation. The mouse model plays a central role for deciphering the underlying mechanisms on a molecular and cellular level. Therefore, we intended to assess in-vivo post-infarct remodeling as comprehensively as possible using an expedient native magnetic resonance imaging (MRI) in the two most prominent infarct models, permanent ligation (PL) of the left anterior descending artery (LAD) versus ischemia reperfusion (I/R). Mice were subjected to either permanent or transient (45 min) occlusion of the LAD. After 3 weeks, examinations were performed with a 7-Tesla small animal MRI system. Data analysis was performed with the freely available software Segment. PL resulted in a massive dilation of the left ventricle, accompanied by hypertrophy of the non-infarcted myocardium and a decline of contractile function. These effects were less pronounced following I/R compared to healthy animals. Single plane assessments were not sufficient to capture the specific differences of left ventricular (LV) properties between the two infarct models. Bulls-eye plots were found to be an ideal tool for qualitative LV wall assessment, whereas a multi-slice sector-based analysis of wall regions is ideal to determine differences in hypertrophy, lateral wall thinning and wall thickening on a quantitative level. We combine the use of polar map-based analysis of LV wall properties with volumetric measurements using simple CINE CMR imaging. Our strategy represents a versatile and easily available tool for serial assessment of the LV during the remodeling process. Our study contributes to a better understanding of the effects of novel therapies targeting the healing of damaged myocardium.

## Introduction

Ischemic heart disease is still the main cause of death worldwide according to the World Health Organization and accounted for more than nine million deaths in 2016^1^. Primary prevention, diagnosis and treatment options of ischemic heart disease have rapidly progressed over the last decades. Particularly timely reperfusion—by both thrombolytic and interventional approaches—reduces myocardial damage dramatically and hence forms the cornerstone of modern therapies for acute ST-segment elevation myocardial infarction (STEMI) patients^2^. Yet, replacement of necrotic myocardium by scar tissue inevitably results in left ventricular remodeling and can eventually lead to heart failure. Left ventricular (LV) remodeling refers to alterations in ventricular architecture and function in response to a variety of forms of myocardial injury and increased wall stress^3,4^. In the context of myocardial infarction, ventricular remodeling is associated with characteristic changes in the early and late post myocardial infarction (MI) phases, respectively. Early LV remodeling results from replacement of necrotic myocardium by fibrotic scar tissue and leads to elongation and thinning of the infarcted zone^5^. Beyond this early phase, LV remodeling is dominated by mechanisms compensating for the loss of vital myocardium. Myocytes in the non-infarcted zone undergo hypertrophic elongation, leading to an increased wall mass, chamber enlargement and a shift from an elliptical to a more spherical chamber configuration^5^. Morphological changes, reduced performance of hypertrophied myocytes and interstitial fibrosis within the non-infarcted zone lead to progressive impairment of LV function.

Initial pivotal studies on LV remodeling were based on post-mortem techniques, such as histopathological tissue analyses^6^. The development of dedicated small animal cardiac magnetic resonance imaging (CMR) initiated a new era, providing a tool which enables high-resolution in-vivo assessment of cardiac function and post-MI remodeling in rodents with utmost precision^7–9^. CMR has become the gold standard for assessing LV remodeling in patients^10,11^ for both clinical studies and optimizing therapy management. Particularly with regards to the development of novel regenerative therapies in translational research, non-invasive assessment of LV remodeling has become an indispensable tool. Preserving cardiac function and anatomical integrity is the ultimate goal of regenerative therapies for ischemic myocardium. Thereby, the mouse is the most important model organism when it comes to testing hypothesized mechanisms of action on a molecular and cellular level^12^. Despite the availability of highly elaborate protocols for CMR based assessment of left ventricular remodeling, such as MR spectroscopy and T1/T2 mapping^13^, CMR-based assessment of left ventricular remodeling in studies aiming at modulating myocardial healing are mostly restricted to volumetric measurements. Yet, novel therapies targeting modulation of the post-MI healing processes and long-term LV remodeling require more comprehensive imaging approaches for gauging LV remodeling. We suppose, that a wider application of T1/T2 mapping and MR spectroscopy is hampered by the difficulty of both acquisition and image analyses and the need for expensive software add-ins. Therefore, we intended to develop a straightforward protocol which is based on native CINE CMR only, but still provides additional information about the specific properties of the remodeled LV wall. Polar maps were applied to describe both morphological and functional wall properties on a qualitative and quantitative level by using the freely available software *Segment*. Thereby, characteristics of the two most commonly used experimental models of myocardial infarction—permanent ligation (PL) of the left anterior descending (LAD) and transient occlusion of the LAD (I/R)—are visualized and assessed. As expected, myocardial damage is significantly more severe in PL compared to I/R resulting in distinct patterns of left ventricular remodeling respectively.

## Methods

### Animal model

129S6/SvEvTac mice were bred in the animal facility of the Rostock University Medical Center according to the German legislation on protection of animals and the Guide for the Care and Use of Laboratory Animals^14^. All experiments performed in this study were approved by the “Landesamt für Landwirtschaft, Lebensmittelsicherheit und Fischerei Mecklenburg-Vorpommern; Rostock, Germany” (registration no. LALLF M-V/TSD/7221.3-1.1-054/15) and carried out in compliance with the ARRIVE guidelines. Myocardial infarctions were induced by either permanent occlusion (PL) or transient occlusion the LAD for 45 min (I/R) as described previously (n = 6 per group)^15,16^. Each animal received an intramyocardial injection of 10 μL PBS mixed with 10 μL Growth Factor Reduced MatrigelTM Matrix. Healthy animals served as a control group (n = 7).

### Cardiac magnetic resonance imaging and analysis

For MRI imaging the mice were anaesthetised with 1.5–2.5% isoflurane in oxygen. Examinations were performed with a 7-T small animal MRI system (BioSpec 70/30, maximum gradient strength 440 mT/m, Bruker BioSpin GmbH, Ettlingen, Germany) using a water-cooled actively shielded gradient system. The MRI system was equipped with a 1H transmit volume coil (86 mm, volume resonator) and a two-by-two receive-only surface coil array (both Bruker BioSpin GmbH). Images of the left ventricle for functional and morphological measurements were acquired using a IntraGate gradient-echo cine sequences (Intragate Cine-FLASH) in five contiguous short-axis planes covering the whole left ventricle following the planning sequences for the short axis view. The following acquisition parameters were used: echo time (TE): 2.38 ms, repetition time (TR): 5.89 ms, flip angle: 15°, 14 frames per cardiac cycle, oversampling: 140, averages: 1, field of view (FOV): 29.4 × 25.2 mm, matrix size: 211 × 180, resolution in-plane: 0.14 × 0.14 mm, slice thickness: 1 mm, scan time per slice: 2 min. Body temperature of the animals was maintained by a water filled heating mat during the whole scan. Temperature of the water-filled heating mat was monitored and controlled by water bath with immersion circulator (HAAKE SC 100 and HAAKE S5P, Thermo Fisher Scientific, Schwerte Germany). Body temperature was continuously monitored by a small rectal temperature probe and maintained between 36 and 37 °C throughout the examination as previously described^17,18^. Respiration cycles and Cardiac BPM were extracted from the Intragate navigator analysis. Mean heart rates during scanning were 340.8 ± 25.4 bpm in the control group, 327.6 ± 19.6 bpm in the I/R group and 343.1 ± 30.3 in the PL group with no significant difference between the groups (*p* = 0.53).

**Figure 1:**
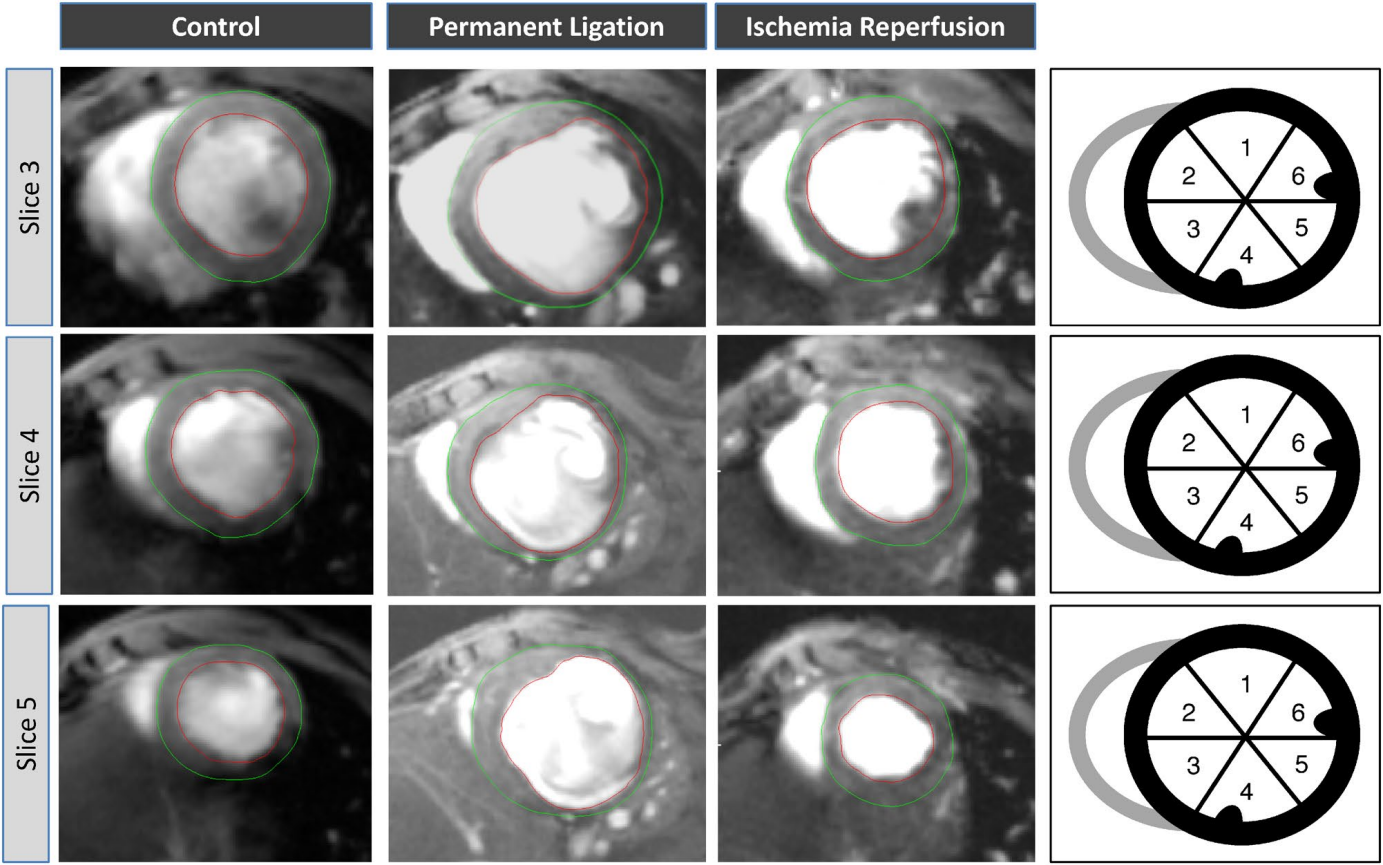
Multi plane assessment of the left ventricular wall. The left ventricle is covered by 5 short-axis slices from basal (1) to apical (5). The scar typically extended from slice 3–5. For regional wall analyses, slices 3–5 were divided into six segments. Septal wall: segment 2, 3. Lateral wall: segment 5, 6.

The freely available software Segment v2.0 R5165 (http://segment.heiberg.sehttp://segment.heiberg.se)^19^ was used for the assessment of cardiac function and morphology. Segmentation of the left ventricle was performed manually. Papillary muscles were excluded when defining endo- and epicardial borders. Definition of end-diastole and end-systole were calculated by the software. Following manual LV segmentation, the software calculated end-diastolic volume (EDV), end-systolic volume (ESV), left ventricular ejection fraction (LVEF), stroke volume (SV) and left ventricular mass (LVM) automatically. Interventricular septal thickness (IVSd), left ventricular end diastolic dimension (LVEDD) and lateral wall thickness (LW) were obtained from the respective end-diastolic mid-ventricular slice. Regional wall analyses were performed on a slice-by-slice basis, according to the user manual. In brief, the left ventricle was covered by 5 short-axis slices from basal (1) to apical (5). Each slice was divided into 6 sectors as visualized in Fig. 1. Qualitative analysis by bulls-eye plots showed that scar typically extended from slice 3–5. Hence, these three slices were included into quantitative regional wall analysis. Multi-plane quantitative analyses of the respective septal and lateral sectors were performed as visualized in Fig. 1.

### Statistics

Normality of data was assessed by the Shapiro–Wilk test. When the normality assumption was met, parametric data of control, PL and I/R group were compared by using one-way analysis of variance (ANOVA). Post-hoc pairwise comparisons were made by the Student–Newmans–Keuls method. When, the normality assumption had been violated, ANOVA on ranks was applied, followed by the Dunn´s Methods for multiple pairwise comparisons. Values of *p* < 0.05 were considered statistically significant. Statistical procedures were performed using *SigmaPlot 11.0*.

### Ethics approval and consent to participate

All experiments performed in this study were approved by the “Landesamt für Landwirtschaft, Lebensmittelsicherheit und Fischerei Mecklenburg-Vorpommern; Rostock, Germany” (registration no. LALLF M-V/TSD/7221.3-1.1-054/15).

### Consent for publication

Not applicable.

## Results

### Volumetric and functional changes

In healthy animals, end-diastolic volume (EDV) was 52.6 ± 4.0 µL whereas the end-systolic volume (ESV) measured 25.7 ± 2.3 µL. Left ventricular ejection fraction (LVEF) was 50.9 ± 1.8% and the stroke volume (SV) 26.9 ± 2.3 µL. Values from healthy animals served as control.

**Figure 2:**
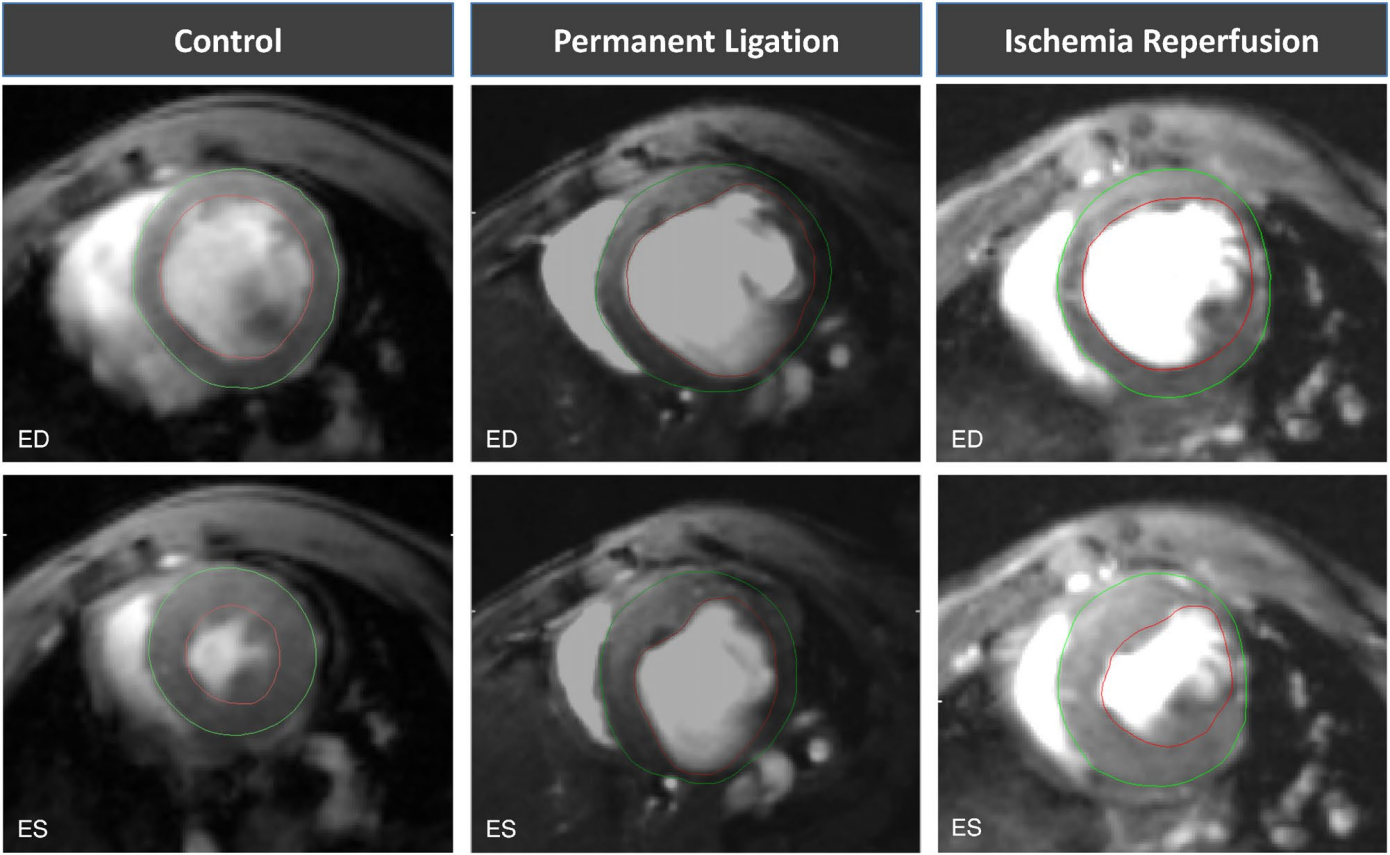
Representative mid-ventricular slices of healthy (control) and infarct animals 3 weeks following MI induction. Upper row: end-diastolic (ED) slices. Lower row: end-systolic (ES) slices. The epicardium is marked with a green line, the endocardium with a red line. Papillary muscles were excluded.

**Figure 3:**
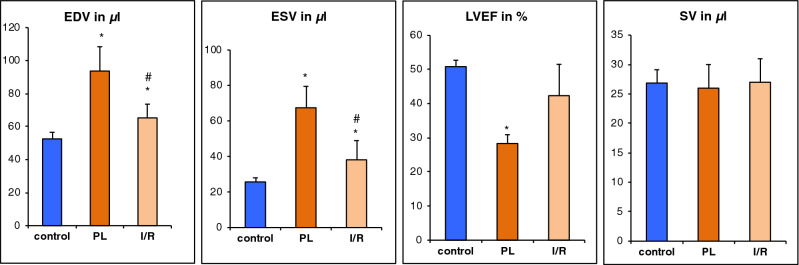
Volumetric and functional changes 3 weeks after MI induction. Values are mean ± SD. *p < 0.05 compared to control group. ^#^*p* < 0.05 compared to PL.

Permanent ligation of the LAD resulted in a massive dilation of the left ventricle with significantly increased diastolic and systolic volumes (EDV: 93.7 ± 14.7 µL, *p* < 0.001 vs. control; ESV: 67.5 ± 12.1 µL, *p* < 0.001 vs. control). These changes in chamber geometry resulted in a dramatic decline of the ejection fraction (LVEF: 28.3 ± 2.6%, *p* < 0.05 vs. control). Yet, the stroke volume remained preserved (SV: 26 ± 4.0 µL, *p* = 0.86) (Figs. 2 and 3).

Transient occlusion (I/R) of the LAD lead to similar, but less severe changes of left ventricular geometry three weeks after MI induction compared to the healthy control group (EDV: 65.3 ± 8.3, *p* < 0.001; ESV: 38.2 ± 10.9 µL, *p* < 0.05) (Figs. 2 and 3). This resulted in a less dramatic reduction of the LVEF (42.3 ± 9.2 µL, *p* > 0.05 vs. control). Similar to the observation in the PL group, the stroke volume remained preserved (SV: 27.0 ± 4.0, *p* = 0.86) (Figs. 2 and 3).

PL resulted in significantly greater volumes of the left ventricle compared to I/R, with a more dramatic decline of the LVEF (Fig. 3).

### Scar thinning and LV hypertrophy

Ventricular remodeling in the early phase post MI is a consequence of the replacement of necrotic myocardium by fibrotic scar tissue, which is subsequently subjected to elongation and thinning. Short-axis parameters of the respective end-diastolic mid-ventricular slice were used with the goal to visualize and quantify this process.

**Figure 4:**
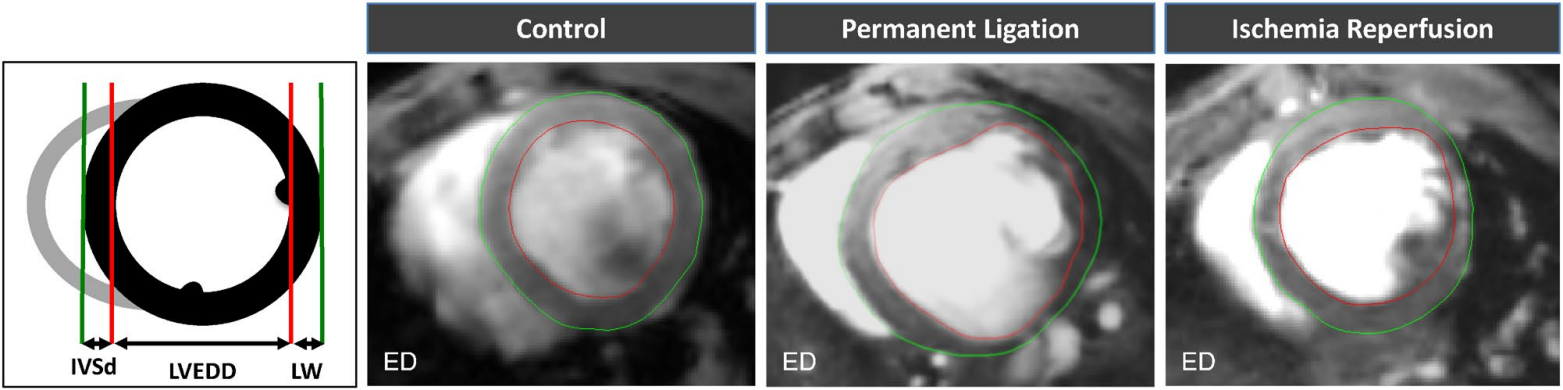
Short-axis End-diastolic mid-ventricular slices—measurement of diastolic interventricular septal thickness (IVSd), LV end diastolic dimension (LVEDD) and lateral wall (LW).

**Figure 5:**
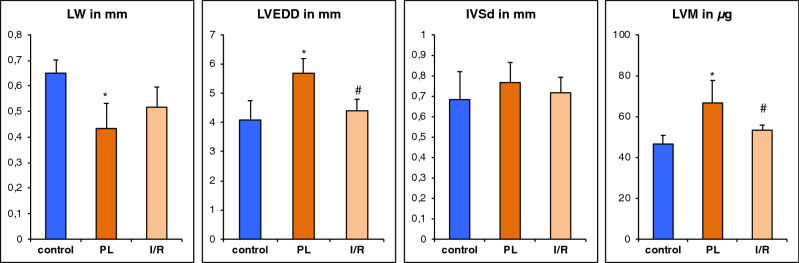
Short-axis end-diastolic dimensions and left ventricular mass. Values are mean ± SD. *p < 0.05 compared to control group. ^#^*p* < 0.05 compared to PL.

Permanent ligation lead to a significant increase of the left ventricular end diastolic dimension compared to the control group (LVEDD: 5.7 ± 0.7 mm vs. 4.1 ± 0.4, *p* < 0.001). The lateral wall thickness (LW) is significantly reduced (LW: 0.43 ± 0.05 mm vs. 0.65 ± 0.08 mm, *p* < 0.05). Yet, LV hypertrophy of the non-infarcted myocardium can only be hypothesized from this single slice assessment of the interventricular septum (IVSd: 0.77 ± 0.14 vs. 0.68 ± 0.08, *p* = 0.29) (Figs. 4 and 5).

Visual and qualitative inspection of the scar area (Fig. 4) reveals a smaller circumferential extent of the scar in the I/R compared the PL group. Whereas LV dilation could be clearly assessed by the determination of EDV and ESV (Fig. 3), single slice assessment of the end-diastolic LV dimension was not significantly increased compared to the control group (LVEDD: 4.4 ± 0.52 mm vs. 4.1 ± 0.40 mm, *p* = 0.26) (Fig. 5). Likewise, scar thinning resulted in a thinner lateral wall—yet not reaching a level of significance—compared to the control group (LW: 0.51 ± 0.10 mm vs. 0.65 ± 0.08 mm, *p* > 0.05). Similar to the PL group, the thickness of the interventricular septum was not significantly increased (IVSd: 0.72 ± 0.01 vs. 0.68 ± 0.08, *p* = 0.29).

Whereas single slice assessment of the IVSd did not sufficiently depict LV hypertrophy, left ventricular mass (LVM) as calculated from all slices covering the left ventricle was significantly increased in the PL group. Yet, the increase of LVM in the I/R group did not reach a level of significance (Fig. 5).

### Regional LV wall analyses

**Figure 6:**
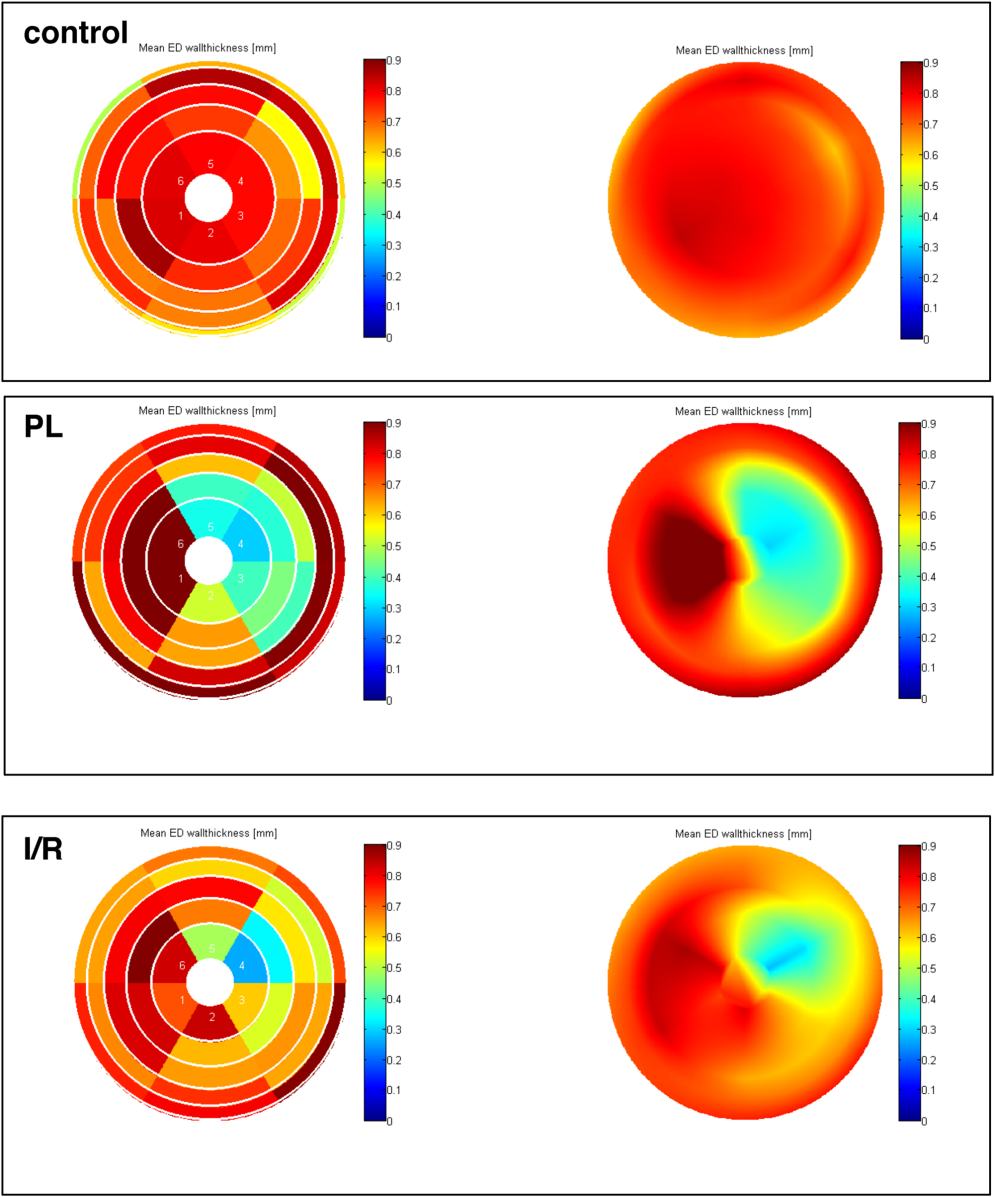
Polar plots of the left ventricle for visual and qualitative assessment of LV wall thickness. Each ring represents one short-axis slice. The apical slice 5 is located in the middle. The basal slice 1 is the outer ring. The “smooth bulls eye view” (right side) visualizes the aneurysm formed in the lateral wall.

For a more comprehensive measurement and visualization of LV hypertrophy of the non-infarcted zone and the wall thinning within the scar area, we performed regional wall analysis of the whole LV (Figs. 1 and 6). Thereby, polar plots showed aneurysm formation within the scar area and a thickened wall in the septal region (Fig. 6). The scar typically extended from the apical to the mid-ventricular slice, corresponding to slice 3–5 (Fig. 6). Each slice was divided into 6 segments as visualized in Fig. 1. The septal wall is represented by the respective segments 2 and 3, the lateral wall by the respective segments 5 and 6.

**Figure 7:**
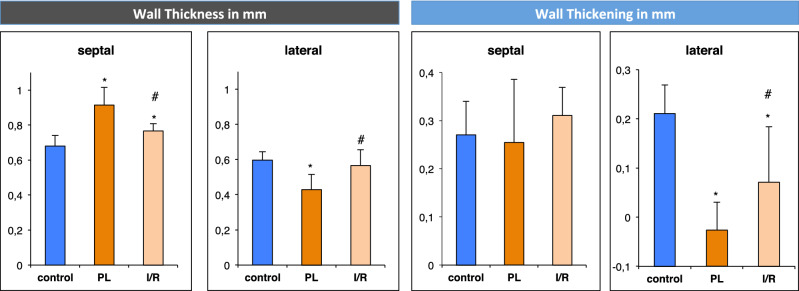
Regional wall analysis of short-axis slices 3–5. Wall thickness was assessed in end-diastolic images. Wall thickening is the difference between end-systolic and end-diastolic wall thickness. Values are mean ± SD. *p < 0.05 compared to control group. ^#^*p* < 0.05 compared to PL group.

Quantitative assessments of regional wall thickness show that hypertrophy of the non-infarcted myocardium is present in both infarct models. Compared to the control group, end-diastolic septal wall thickness is significantly increased both in the PL group (0.91 ± 0.10 mm vs. 0.68 ± 0.06 mm, *p* < 0.0001) and the I/R group (0.77 ± 0.04 mm vs. 0.68 ± 0.06 mm, *p* < 0.005) (Fig. 7).

Thinning of the infarct zone was assessed by measuring the end-diastolic lateral wall thickness (segments 5, 6). Permanent ligation of the LAD resulted in a significant thinning of the lateral wall compared to the control group (0.43 ± 0.09 mm vs. 0.60 ± 0.05, *p* < 0.001). Transient occlusion potentially resulted in a slight, yet not significant reduction of the lateral wall thickness (0.57 ± 0.09 vs. 0.60 ± 0.05 mm, *p* = 0.32).

**Figure 8:**
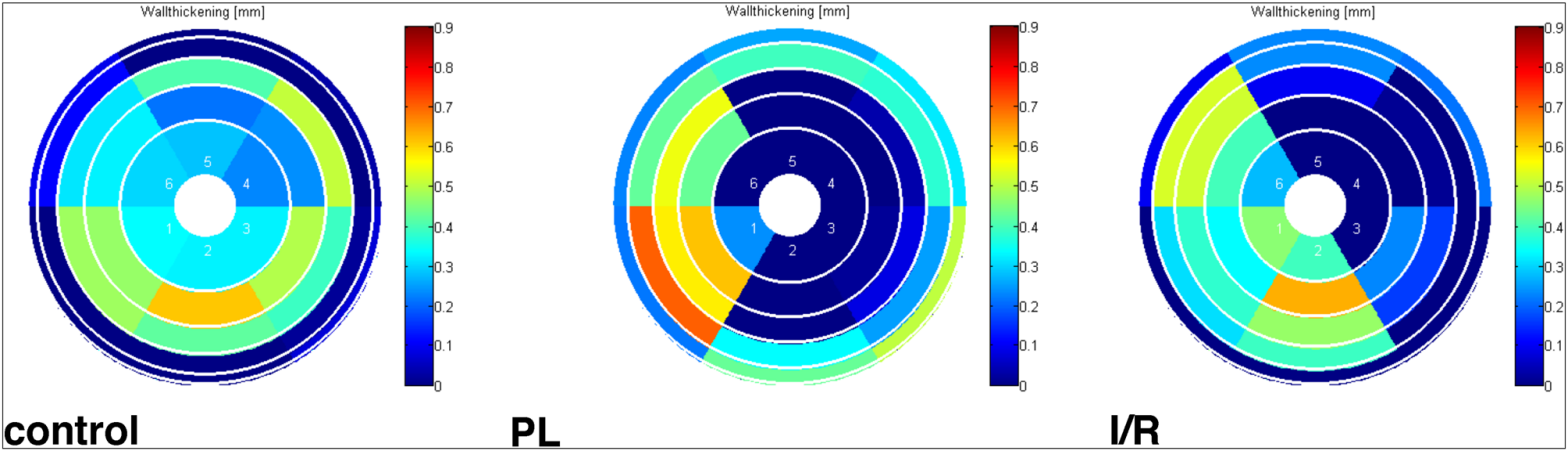
Left ventricular wall thickening. Representative polar maps of visualizing LV wall thickening of healthy (control) and MI animals.

Assessment of the regional wall thickness was used as surrogate measures of LV hypertrophy and scar thinning. Both parameters are determined from end-diastolic images and represent morphological measures. Yet, we assumed quality and composition of the scar area would affect radial wall motion properties. Therefore, wall thickening—the difference between end-diastolic and end-systolic wall thicknesses—was determined for both infarct models compared to the control group. Permanent ligation of the LAD led to a significantly reduced wall thickening in the infarct region (− 0.03 ± 0.06 mm vs. 0.21 ± 0.06 mm, *p* < 0.001) but preserved wall thickening in the septal region (0.25 ± 0.13 mm vs. 0.27 ± 0.07 mm, *p* = 0.31). Similarly, transient occlusion of the LAD resulted in significantly reduced wall thickening in the infarct region (0.07 ± 0.11 mm vs. 0.21 ± 0.06 mm, *p* < 0.005) with preserved septal wall thickening (0.31 ± 0.06 mm vs. 27 ± 0.07 mm, *p* = 0.31) (Figs. 7 and 8).

## Discussion

The size of a myocardial infarction has been correlated with the extent of LV remodeling in the late 70s^20^ in a rat model and eventually been confirmed in patients in the 90s^21^. LV remodeling is a progressive process, which is closely linked to activation of the renin–angiotensin–aldosterone axis (RAAS) and the adrenergic nervous system. Pharmacological inhibition of these mechanisms has the potential to stop or even reverse pathological remodeling^5^. The imaging-based parameters EDV, ESV and LVEF proved to be reliable surrogate parameters to both estimate cardiovascular events and the effects of pharmacological RAAS inhibition^5^. Hence, monitoring infarct size and left ventricular chamber dimensions are well-established surrogate markers for the effect of therapies for the treatment of myocardial infarctions in patients.

Novel therapies aiming at reducing myocardial damage or restoring contractile function usually target very specific biological mechanisms. Therefore, pilot proof-of-concept studies are most commonly conducted in animal models. Mere global volumetric and functional parameters will not suffice to capture these effects. From a translational perspective, development of novel therapies targeted at modulating myocardial healing requires methods for sequential and comprehensive in-vivo assessment of the remodeling process.

Interestingly, a variety of highly elaborate protocols for CMR based assessment of left ventricular remodeling^13^ have been developed. Yet, studies aiming at modulating myocardial healing are mostly restricted to volumetric measurements. The application of elaborate CMR techniques, e.g. MR spectroscopy, requires elaborate and expensive software and profound expertise in CMR radiology, which might hamper a wider application of these attractive tools. This motivated us to develop a protocol, which is based on CINE CMR only, but still provides additional and highly valuable information about the specific properties of the remodeled LV wall.

Therefore, mice were subjected to permanent occlusion (PL) or transient occlusion (I/R) of the LAD, respectively. Post-infarct ventricular remodeling was assessed after 3 weeks by native CMR. PL resulted in a massive dilation of the left ventricle and thinning of the scar, with consecutive formation of an anterolateral aneurysm. Interestingly, despite dramatic increases in EDV and ESV and an according decrease of the LVEF, stroke volume (SV) remained unaffected (Fig. 3). Compared to the PL group, myocardial damage induced by I/R was less pronounced, accordingly LV remodeling was significantly less dramatic. Similar to the PL group, stroke volume was preserved.

Our overall observation, that LV dilation and consecutive impairment of cardiac function parameters caused by PL is more pronounced compared to I/R, is well in line with findings by other groups^17,22–24^. The observation that SV remains preserved despite LV dilation in both infarct models has also been made by Soepriatna et al. in mice^17^. Similar findings in patients, namely the fact that SV is commonly not reduced despite enormous LV dilation in patients, has been extensively reviewed by Maciver et al.^25^ and can most probably be explained by a compensated stage of heart failure in both animals and patients at the time of assessment.

Post-infarct LV remodeling comprises characteristic alterations in both infarcted and non-infarcted myocardium. Fibrotic repair of the necrotic area results in scar formation with consecutive elongation and thinning of the infarcted zone^5^. On the other hand, adaptive mechanisms, such as hypertrophic myocyte elongation in the non-infarcted myocardium and a consecutive increase of LV mass have the capacity to compensate for the loss of functional cardiomyocytes to a certain extent^5^. These adaptive mechanisms retain normal cardiac output in the initial post-MI phase but eventually result in progressive fibrosis of the pathologically hypertrophied non-infarcted myocardium, chamber enlargement and a decline in ventricular performance^5^.

PL of the LAD induces a large transmural scar with a thin remaining rim of endocardial-located viable cardiomyocytes resulting in progressive thinning of the fibrous scar^26,27^. In contrast, I/R induces a longitudinal scar layer in the middle of the LV myocardium with non-infarcted endocardial and epicardial myocardium left^27^. As a consequence, LV wall thickness in the infarct region is maintained. Yet, both infarct models show signs of compensatory hypertrophy of the non-infarcted region measured by histological methods^24^.

Differences in regional left ventricular wall properties between PL and I/R in mice had been described mostly in post mortem studies until recently^26–29^. The evolution of elaborate techniques for qualitative tissue assessment resulted in studies describing differences in LV wall properties between the two infarct models by both echocardiographic-based regional myocardial strain analysis^17^ and CMR-based T1/T2 mapping^23^.

Here, we developed a simple and straightforward protocol, based on CINE CMR only, to provide a tool for regional LV wall analysis for scientists in the field of experimental cardiology who are not designated experts in the field of cardiovascular imaging.

In our study, we measured changes of LV wall dimensions by both single plane and multiple plane approaches. Thereby, using a clinically well-established single plane view, the mid-ventricular parasternal short axis, only thinning of the infarct wall reached the level of significance. Hence, we used a multi-slice approach for more comprehensive analysis of the LV wall properties in both infarct models (Fig. 1). We used left ventricular mass (LVM) as a surrogate for overall hypertrophy of the non-infarcted myocardium. LVM was significantly increased by PL and to a less but significant extent by I/R (Fig. 3). Bulls-eye plots were used for qualitative assessment of the specific remodeling patterns of PL and I/R respectively. We used a multi-plane approach to capture differences of septal and lateral wall properties as visualized in Fig. 1.

We found PL to induce dramatic thinning of the lateral wall accompanied by hypertrophy of septal wall regions, a finding, which is in line with previous reports^24,26^. In contrast to single plane approaches^26^, we found I/R to result in LV hypertrophy and a consecutive increase in septal wall thickness in our study. Yet, as supposed, lateral wall thickness was preserved after I/R.

For functional assessment of the scarred myocardium, we measured regional wall thickening in both infarcted and non-infarcted regions. As one would expect, the dilated scar in PL mice exhibited a negative thickening value, which might represent the passive systolic expansion of the aneurysmal lateral wall. Interestingly, despite retained thickness of the lateral wall after I/R, wall thickening was dramatically reduced. This finding suggests, that scarring after I/R has significant implications on LV function, despite a lack of changes on lateral wall dimensions. Similarly, Soepriatna et al. who used echocardiography based three-dimensional strain mapping, found significantly higher strain values within the infarcted myocardium of I/R compared PL mice^17^. Haberkorn et al. also described distinct differences in T1 values within the infarct region between IR and PL in mice, which they ascribe to fibrotic changes^23^.

## Conclusions

By the use of multi-plane sector analysis, we succeeded in capturing both morphological and functional differences between PL and I/R regarding post-infarction LV remodeling by native CMR. We believe our approach to offer a deeper understanding of the specific functional implications of both PL and I/R. This will provide an excellent and simple tool for unraveling the effects of novel therapies targeting at healing damaged myocardium.

### Limitations

We did not apply late gadolinium enhancement CMR, which represents the gold standard for determination of circumferential scar size extent. The scarred area can only be estimated indirectly based on the “thinning” of the infarct region. Furthermore, our approach is based on the repeated assessment of five contiguous 1 mm thick short-axis slices. Hence, the longitudinal increase in LV mass and wall motion properties may have been missed. Also, the heart rates below 400 per minute, as a potential result of anesthesia, may have affected the assessed functional parameters^30^. Lack of intra- and inter-observer analysis may also have produced some inaccuracy. Finally, the injection of Matrigel matrix into the infarct region of all mice may have contributed to our failure to detect significant wall-thinning in the infarct regions of the I/R mice.

## Data Availability

The datasets used and analyzed during the current study are available from the corresponding author on reasonable request.

## Abbreviations

LAD: Left anterior descending artery
MRI: Magnetic resonance imaging
PL: Permanent ligation
I/R: Ischemia reperfusion
LV: Left ventricle
STEMI: ST-segment elevation myocardial infarction
MI: Myocardial infarction
CMR: Cardiac magnetic resonance imaging
EDV: End-diastolic volume
ESV: End-systolic volume
LVEF: Left ventricular ejection fraction
SV: Stroke volume
LVM: Left ventricular mass
IVSd: Interventricular septal thickness
LVEDD: Left ventricular end diastolic dimension
LW: Lateral wall thickness
SD: Standard deviation
ED: End diastolic
ES: End systolic
